# Role of Leptin in Cardiovascular Diseases

**DOI:** 10.3389/fendo.2020.00354

**Published:** 2020-06-16

**Authors:** Mareike S. Poetsch, Anna Strano, Kaomei Guan

**Affiliations:** Medical Faculty Carl Gustav Carus, Institute of Pharmacology and Toxicology, Technische Universität Dresden, Dresden, Germany

**Keywords:** leptin receptor, leptin resistance, metabolism, cardiovascular disease, obesity, type 2 diabetes mellitus

## Abstract

The adipocyte-derived adipokine leptin exerts pleiotropic effects, which are essential for the regulation of energy balance and cell metabolism, for controlling inflammatory and immune responses, and for the maintenance of homeostasis of the cardiovascular system. Leptin resistance in obese or type 2 diabetes mellitus (T2DM) patients is defined as a decrease in tissue response to leptin. In the cardiovascular system, leptin resistance exhibits the adverse effect on the heart's response to stress conditions and promoting cardiac remodeling due to impaired cardiac metabolism, increased fibrosis, vascular dysfunction, and enhanced inflammation. Leptin resistance or leptin signaling deficiency results in the risk increase of cardiac dysfunction and heart failure, which is a leading cause of obesity- and T2DM-related morbidity and mortality. Animal studies using leptin- and leptin receptor- (Lepr) deficient rodents have provided many useful insights into the underlying molecular and pathophysiological mechanisms of obese- and T2DM-associated metabolic and cardiovascular diseases. However, none of the animal models used so far can fully recapitulate the phenotypes of patients with obese or T2DM. Therefore, the role of leptin in the human cardiovascular system, and whether leptin affects cardiac function directly or acts through a leptin-regulated neurohumoral pathway, remain elusive. As the prevalence of obesity and diabetes is continuously increasing, strategies are needed to develop and apply human cell-based models to better understand the precise role of leptin directly in different cardiac cell types and to overcome the existing translational barriers. The purpose of this review is to discuss the mechanisms associated with leptin signaling deficiency or leptin resistance in the development of metabolic and cardiovascular diseases. We analyzed and comprehensively addressed substantial findings in pathophysiological mechanisms in commonly used leptin- or Lepr-deficient rodent models and highlighted the differences between rodents and humans. This may open up new strategies to develop directly and reliably applicable models, which resemble the human pathophysiology in order to advance health care management of obesity- and T2DM-related cardiovascular complications.

## Background

Recent reports of the world health organization (WHO) emphasize the dramatic increase in the prevalence of obesity, which has nearly tripled since 1975. In 2016, almost 39% (1.9 billion) of adults worldwide were overweight and 13% (>650 million) were obese. The prevalence of obesity in children and young adolescents (aged 5–19 years) has also risen from 4% in 1975 to 18% in 2016 (1). A common co-morbidity of excess bodyweight and physical inactivity is type 2 diabetes mellitus (T2DM), which accounts for 90–95% of all diagnosed diabetes cases in adults (2). The global prevalence of diabetes among adults has risen from 4.7% in 1980 to 8.5% in 2014 and by 2014 diabetes mellitus affected more than 422 million people. These escalating healthcare problems of monumental proportions represent a serious hazard of developing cardiovascular diseases with significant morbidity and mortality (2). However, the complex mechanisms underlying the detrimental impact of obesity and diabetes on the cardiovascular system are poorly understood (3). In the last two decades, many studies show that the adipocyte-derived peptide hormone leptin plays an important role in linking obesity, inflammation, metabolic syndrome, and cardiovascular diseases.

Since the discovery of leptin in 1994 (4), extensive research in various murine model systems revealed a complex interplay of genes and environmental factors involved in the development of obesity, which impacts homeostatic control of energy metabolism and feeding in the central nervous system [reviewed in (5)]. As a circulating adipokine, leptin binds to the leptin receptor (LEPR, also known as OB-R) in the arcuate nucleus (ARC) of the hypothalamus where it functions as an anorexigenic (appetite-suppressing) peptide hormone that regulates food intake and energy expenditure. Two types of neurons in the ARC with antagonistic functions are major targets of leptin: (i) pro-opiomelanocortin (POMC) neurons and (ii) neuropeptide-Y (NPY) and agouti-related peptide (AgRP) neurons. In the hypothalamus, both POMC and NPY/AgRP neurons are also major targets of insulin, therefore, the actions of leptin and insulin are interconnected and contribute to optimal metabolic control. While insulin maintains adequate energy storage and utilization, leptin reduces continuous energy intake. It is known that leptin and insulin act on different subpopulations of POMC neurons (6) and both leptin and insulin are required for and play key roles in the central regulation of energy expenditure and glucose homeostasis. Although leptin and insulin are two of the most widely studied peptide hormones, new studies to decipher their precise functions are urgently needed. Recently, a study revealed that leptin increases the expression of the insulin receptor phosphatase PTP1B in the ARC, which leads to reduced insulin sensitivity and impaired insulin-mediated suppression of hepatic glucose production in diet-induced obese mice (7). The central pharmacological inhibition or ARC-targeted deletion of PTP1B restores insulin-mediated suppression of hepatic glucose production in obese mice (7). Moreover, another recent study has shown that hyperleptinemia is a driving force for obesity and its associated metabolic syndrome and that partial deletion, rather than complete elimination of leptin restores hypothalamic leptin sensitivity and increases insulin sensitivity in diet-induced obese mice (8).

This review is focused on leptin and LEPR signaling in the (dys-)regulation of the cardiovascular system in the context of obesity and metabolic syndrome. Since most of the molecular insights regarding leptin signaling are derived from studies in rodent models, we are aiming to highlight the differences in the leptin-LEPR interaction in rodents and humans. Finally, we provide an outlook on recent perspectives on deciphering the underlying pathophysiological mechanisms of leptin-mediated cardiomyopathy and future therapeutic approaches for the treatment of diabetes-mediated cardiovascular disease.

## Leptin and its Receptors

Leptin is primarily produced by adipocytes and released from small vesicles within adipocytes in a diurnal pulsatile manner, with higher rates in the evening and early morning. Although the release of leptin occurs independent of its mRNA regulation (9), increase of leptin mRNA transcription is necessary to retain constant rates of leptin secretion and to prevent rapid exhaustion of leptin vesicle stores (10). Once secreted, leptin circulates in the serum both in a free and in a bound form.

The leptin receptor (LEPR, or OB-R) was identified as a single membrane-spanning receptor that structurally resembles class I cytokine receptors. Interestingly, the gene encoding the leptin receptor, both in mice (*db*) and in humans (*LEPR*), comprises a dual promoter: while the *B219/OB-R* promoter generates *db/LEPR* transcripts only, the *OB-RGRP* promoter initiates transcription of both *db/LEPR* and *OB-RGRP/LEPROT* (leptin receptor gene-related protein/leptin receptor overlapping transcript) genes (11). LEPR is mainly localized in intracellular compartments, including endosomes, the trans-Golgi apparatus, or the endoplasmic reticulum, and to a lesser extent on the plasma membrane (12). Studies revealed that OB-RGRP/LEPROT (also known as endospanin-1) negatively affected the expression of LEPR on the cell surface (13).

**Figure 1:**
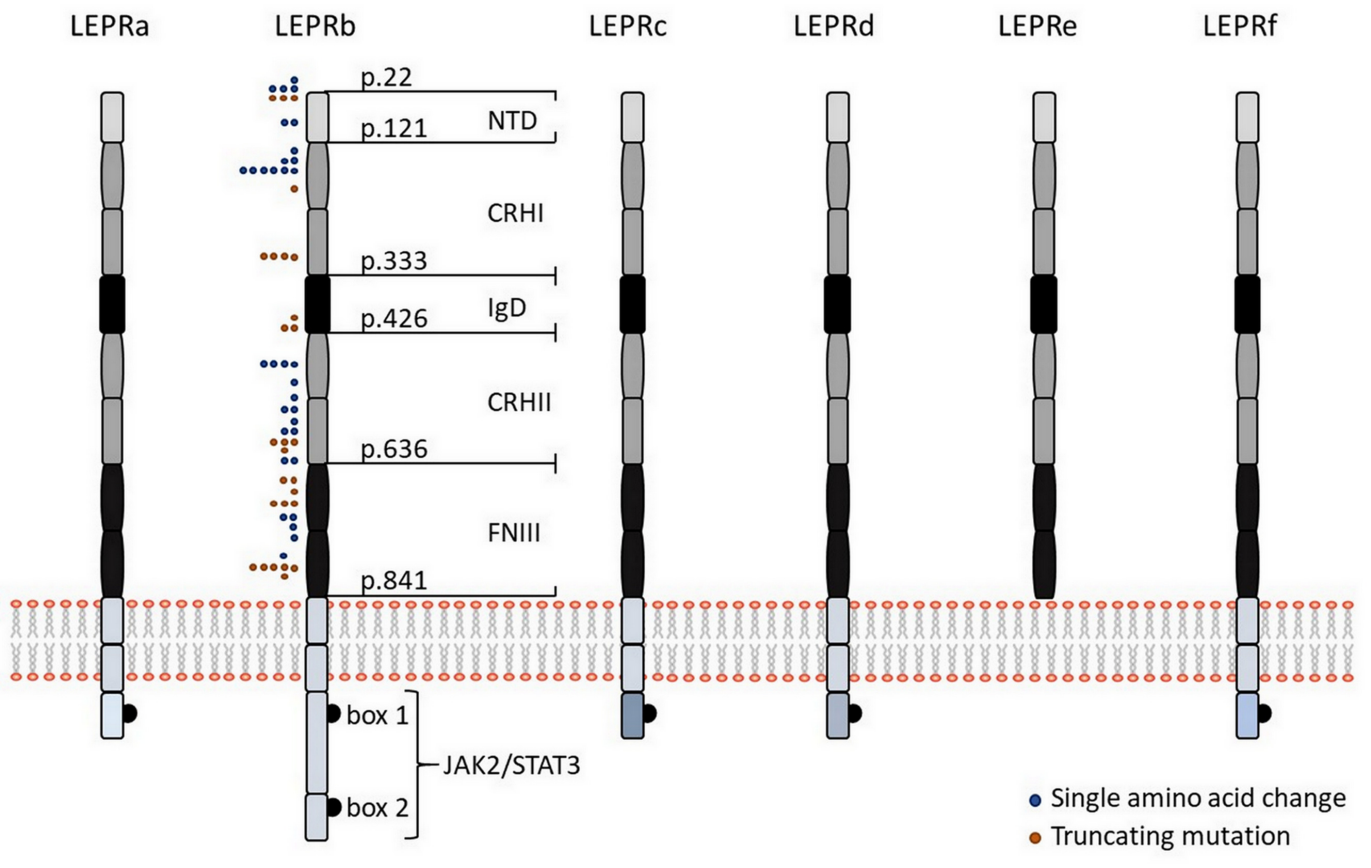
Leptin receptor isoforms and visualization of mutations in the human LEPR protein. Schematic representation of the six different isoforms of LEPR in humans (LEPRa, b, c, d, e, and f). All isoforms share identical extracellular domains as well as the first 29 amino acids containing box 1 motif for binding of JAK2 of the intracellular domain but they differ in the length and sequence of the C-terminal domain. The intracellular domain of LEPRb contains another JAK binding domain (“box 2”) in addition to a STAT binding site, making LEPRb the predominant isoform responsible for signal transduction. Five of the six isoforms have a transmembrane domain and are generated by alternative mRNA splicing, while the shortest isoform LEPRe is derived by ectodomain shedding at the membrane-spanning domain. Colored dots indicate positions of human LEPR mutations, which result in single amino acid changes (blue dots), or a truncated protein (orange dots), as previously described (38). NTD, N-terminal domain of undefined function; CRHI and CRHII, cytokine receptor homologous domain I and II; IgD, immunoglobulin-like domain; FNIII, fibronectin type 3 domains; JAK2, Janus family tyrosine kinase 2; STAT3, signal transducer and activator of transcription 3.

Six isoforms of LEPR (LEPRa, b, c, d, e, and f, also known as OB-Ra, b, c, d, e, and f) with distinct biological activities have been identified (Figure 1). In the mouse, all of them are generated by alternative mRNA splicing (14). In humans, alternative splicing also generates distinct LEPR isoforms with the exception of the soluble isoform LEPRe, which is derived from membrane-spanning receptors by ectodomain shedding (15). All LEPR isoforms share an identical, highly glycosylated extracellular domain of 840 amino acids, which comprises six subdomains: a N-terminal domain of undefined function (NTD), two cytokine receptor homologous domains (CRHI, CRHII) harboring a Trp-Ser-X-Trp-Ser motif, an immunoglobulin-like domain (IgD), and two fibronectin type 3 domains (FNIII). Leptin uses multiple binding sites in the CRHII and IgD domains to engage LEPR, which are fundamental for leptin-binding and LEPR activation (Figure 1).

The membrane-bound isoforms LEPRa, b, c, d, and f comprise the same sequence of a 23-amino acid spanning trans-membrane domain as well as the first 29 amino acids containing box 1 motif of the intracellular domain, which is required for binding of Janus family tyrosine kinase 2 (JAK2). Membrane-bound isoforms only differ in the length of the intracellular domains. LEPRa, c, d, and f comprise short intracellular tails of 32–40 amino acids with unique C-termini and are crucial for internalization and lysosomal-dependent degradation of leptin (16). In contrast, the intracellular domain of LEPRb comprises ~306 amino acids (shorter in mice than in humans) and contains another JAK binding domain (“box 2”) in addition to a STAT binding site, making LEPRb the predominant isoform responsible for signal transduction (17). Binding of leptin to LEPRb results in a conformational change that promotes receptor homo-oligomerization and activation of JAK2 through auto-phosphorylation (18). Activated JAK2 phosphorylates LEPRb at three different tyrosine residues (Tyr^985^, Tyr^1077^, Tyr^1138^), each comprising a Src homology 2 (SH2)-binding motif (19–21). Phosphorylation of Tyr^1077^ promotes recruitment and activation of STAT5 (20, 21), whereas phosphorylation on Tyr^1138^ results in recruitment and activation of STAT1/5 and STAT3, which permit auto-phosphorylation of STAT proteins (20). Phosphorylated STAT3 dimerizes and translocates to the nucleus to activate transcription of target genes, including suppressor of cytokine signaling 3 (SOCS3), as a negative regulator of LEPRb-induced JAK/STAT signaling. Phosphorylation of Tyr^985^ likewise activates SOCS3 and thereby inhibits phosphorylation of LEPRb (19). In addition, leptin-mediated activation of LEPRb-associated JAK2 promotes activation of the phosphatidylinositol 3-kinase (PI3K)/AKT signaling pathway by recruiting the insulin receptor substrates IRS1 and IRS2, which are key molecules for regulation of glucose homeostasis, lipid metabolism, protein synthesis as well as cell proliferation and survival (22).

The soluble LEPRe isoform, which lacks a trans-membrane domain, circulates in the blood as dimers or oligomers. LEPRe functions as a serum-binding protein that transports leptin across the blood-brain barrier (23). Furthermore, LEPRe modulates steady-state leptin levels and leptin bioactivity by complexing free leptin, which delays its degradation and clearance (24).

## Leptin in Obesity and Type 2 Diabetes Mellitus

Congenital leptin deficiency is a form of monogenic obesity and caused by mutations in the gene encoding leptin (*LEP*, known as the *ob* gene in the mouse). Patients with homozygous *LEP* mutations show undetectable levels of leptin in the serum, are characterized by severe early-onset obesity and marked hyperphagia, and develop glucose intolerance and insulin resistance (25). As leptin has a direct effect on insulin sensitivity, leptin replacement therapy was proposed as a novel therapeutic option in metabolic disorders, including insulin resistance and T2DM. In fact, leptin replacement successfully normalized or improved most of the phenotypes in patients with congenital leptin deficiency, including decrease in insulin resistance, improvement of lipid profile, and remarkable weight loss (25). The leptin-deficient *ob*/*ob* mouse is a widely used model to study obesity and largely resembles congenital leptin deficiency in humans. *Ob/ob* mice have a homozygous nonsense mutation (C to T) in the *ob* gene, which causes premature termination of leptin translation (R105X). As a result, although there are high levels of leptin mRNA in adipocytes, *ob/ob* mice completely lack functional leptin (4). Similar to patients with congenital leptin deficiency, *ob/ob* mice are characterized by hyperphagia, hyperinsulinemia, insulin resistance, and massively increased amounts of white adipose tissue. Exogenous leptin application almost completely reversed these phenotypes. However, as insulin release capacity remains high throughout life and hyperglycemia is reduced after 6 months of age, the *ob*/*ob* mouse model is probably not the best study model to cover all manifests of T2DM in humans (26, 27).

In humans, serum leptin levels positively correlate with body mass index (BMI), percentage of body fat, fat mass, and size of adipocytes (28). Obesity-associated enlargement of adipocytes in humans results in accelerated secretion of leptin and therefore higher serum leptin levels, which may also result from chronic hyperinsulinemia and increased cortisol turnover. Additionally, many factors such as free fatty acids, estrogen, tumor necrosis factor-α (TNF-α), or impaired renal clearance, are known to stimulate leptin secretion (29). Previous studies have also shown that hormones and the nutritional status influence serum leptin levels, independent of obesity. Leptin levels usually increase 4–7 h after meal consumption, mainly as a result of insulin-stimulated glucose utilization, leading to up-regulation of mRNA transcription and release of leptin from subcutaneous and omental adipose tissue (29). Similar, glucocorticoids increase leptin mRNA and plasma leptin levels, but as they cause insulin resistance and hyperinsulinemia, the effect of glucocorticoids on leptin levels may be indirect (30). In contrast, low insulin and glucose levels, in concert with elevated levels of catecholamines, are known to decrease serum leptin levels in response to conditions of fasting or starvation (29). In addition, other factors, such as exposure to cold (31), activation of β_3_-adrenergic receptors (32), and elevated intracellular cAMP levels (33) have been shown to inhibit leptin secretion and expression.

The majority of obese patients are hyperleptinaemic. High levels of leptin are associated with insulin resistance, hypothalamic inflammation, and disturbances in hemostatic factors, which are all risk factors for the development of hypertension, metabolic syndrome, or other cardiovascular diseases (34). Opposite to the benefits of leptin replacement therapy in patients with congenital leptin deficiency, treatment of common obese patients with recombinant leptin did not result in reduced food intake and weight loss (25), leading to the spread of the concept of leptin resistance. Leptin resistance is a condition by which hypothalamic neurons are less sensitive to leptin or do not respond to leptin despite the presence of copious amounts of leptin (35). Mechanisms underlying the development of leptin resistance have been discussed previously [reviewed in (35–37)]. Leptin resistance is mainly caused by mutations in the genes encoding leptin and LEPR, reduced expression of LEPR at the plasma membrane level, deterioration of LEPR function and signaling, or alterations in the transport of leptin across the blood-brain barrier (35–37). Furthermore, elevated leptin levels increase the predisposition of patients to diet-induced obesity, which results in a vicious circle leading to a further increase in leptin levels and aggravation of existing leptin resistance, indicating that leptin itself plays an important role in the development of its resistance, as termed “leptin-induced leptin resistance” (37). Importantly, two recent studies have proposed that hyperleptinemia-associated leptin resistance is indeed a driving force for obesity and its associated various metabolic disorders, suggesting that new therapeutic tools to restore leptin sensitivity could offer a new strategy to promote weight loss in obese patients and to treat obesity-associated complications (7, 8).

Obesity-causing LEPR mutations are usually inherited in an autosomal-recessive manner. The majority of LEPR mutations have been reported in populations in which consanguineous marriage is common. Patients carrying a LEPR mutation are hyperphagic and show early-onset morbid obesity. To date, 57 cases with 38 distinct LEPR mutations have been described with most mutations occurring in the extracellular domain, particularly in the leptin-binding or the LEPR activation domain (Figure 1). Not a single LEPR mutation has been identified in the intracellular domain (38). The first LEPR mutation linked to severe obesity was described as a G to A transversion in the splice site of exon 16 (c.2597 +1G>A), which results in the exon 16 skipping and premature termination of protein synthesis (39). Functional *in silico* analyses are available for 35 LEPR mutations, but functional *in vitro* data have only been described for four mutations (38, 40). Within these four mutations, only patients carrying the compound heterozygous LEPR mutation R612H (together with a non-sense mutation) revealed residual ability to phosphorylate STAT3 in response to leptin. The missense mutations A409E, W664R, and H684P caused a complete loss of leptin signaling. However, all patients with these four mutations were characterized by hyperphagia, severe early-onset obesity, alterations in immune function, and delayed puberty (40). In future, functional *in vitro* and/or *in vivo* analyses of other LEPR mutations are essential to assess the genotype and phenotype correlation, which will help to understand the precise regulation of leptin signaling.

Obesity-causing LEPR mutations have also been linked to insulin resistance and development of T2DM, which due to its slow progression, often remains asymptomatic for many years. In fact, all patients carrying an obesity-causing LEPR mutation (aged 4 to 55 years) show hyperinsulinemia to an extent consistent with the degree of obesity, but T2DM was only reported in two adults (41- and 55-year-old) (40). These data, together with other published data in humans, indicate that subjects with obesity-causing LEPR mutations may be at high risk for early-onset insulin resistance and T2DM (38).

Lepr-deficient *db*/*db* mice are one of the most useful models to study the pathophysiology of obesity-associated T2DM. *Db/db* mice display an autosomal recessive G>T mutation at Arg 890 in the *db* gene, which results in abnormal splicing and the replacement of the full-length leptin receptor (Ob-Rb) by Ob-Ra (14). As Ob-Ra lacks the most part of the cytoplasmic domain, leptin-mediated signal transduction is severely impaired in *db/db* mice. Similar to *ob*/*ob* mice, homozygous *db*/*db* mice display severe and early-onset obesity, associated with severe hyperinsulinemia, but normal glucose tolerance by 4 weeks of age. By 8 weeks of age, *db/db* mice develop insulin resistance and hyperglycemia. However, *db*/*db* mice cannot fully recapitulate the obesogenic phenotype of T2DM patients, which are characterized by elevated triglycerides and very low-density lipoprotein (VLDL) cholesterol, normal or even increased low-density lipoprotein (LDL) cholesterol and total cholesterol, but reduced high-density lipoprotein (HDL) cholesterol. In contrast, *db*/*db* mice show elevated cholesterol levels due to an increase in HDL cholesterol (41).

Similar to mouse models, Zucker fatty (*fa*/*fa*) rats and Zucker diabetic fatty (ZDF) rats exhibit overt obesity by 5 weeks of age. The *fa*/*fa* rat harbors the homozygous missense mutation Q269P in the CRH1 domain in all Lepr isoforms, leading to loss of receptor function due to defective Lepr dimerization. The ZDF rat is an inbred sub-strain of the *fa*/*fa* rat, which, in addition to the Lepr mutation, carries an inherited autosomal recessive genetic defect in β-cell transcription. Phenotypically, both *fa*/*fa* and ZDF rats show consistently elevated levels of LDL and HDL cholesterol as well as increased activity of lipoprotein lipase, which are different from those of T2DM patients. Additionally, both transgenic rat models develop insulin resistance at different age. However, *fa*/*fa* rats are not hyperglycemic although they reveal hyperinsulinemia at 3–4 weeks of age, but plasma insulin levels return to normal by 30 weeks of age. In contrast, plasma insulin levels of ZDF rats dramatically increase from 6 to 8 weeks of age and decline afterwards. ZDF rats become insulinopenic (insufficient secretion of insulin) at 14 weeks of age due to pancreatic β-cell insufficiency and the male rats develop gender-specific hyperglycemia by 10–12 weeks of age (27, 42).

## Leptin, Inflammation, and the Immune System

Studies have shown that obesity is associated with chronic low-grade systemic inflammation, which results from changes in both the innate and the adaptive immune system (43, 44). In children and adults who are overweight or obese, the inflammatory state was reflected by elevated levels of circulating pro-inflammatory cytokines, including TNF-α, interleukin (IL)-6, and C-reactive protein, and by the activation of pro-inflammatory signaling pathways in the adipose tissue. Higher concentrations of pro-inflammatory cytokines have also been detected in individuals who are insulin resistant and obese and are used to predict the development of T2DM and cardiovascular diseases (43, 45). Leptin production is regulated by inflammatory stimuli, including lipopolysaccharide, TNF-α, IL-6, and IL-1β, and plasma leptin levels increase during acute infection, inflammation, or sepsis. LEPR has been detected in all cell types of innate and adaptive immunity, including macrophages, dendritic cells, nature killer (NK) cells, neutrophils, and lymphocytes. Leptin modulates the immune response and inflammation at various levels, which have been detailed reviewed previously (46, 47) and are discussed here only briefly.

Leptin exerts various functions in the innate immune system. In monocytes, leptin stimulates the release of pro-inflammatory cytokines, including TNF-α, IL-6, and IL-12 and induces expression of cell surface markers important for activation of resting monocytes. It promotes proliferation and differentiation of monocytes into macrophages, but on macrophages, leptin acts as an anti-apoptotic cytokine by stimulating phagocytosis. Moreover, leptin stimulates chemotactic activity and function of neutrophils by enhancing production of oxygen species crucial for host cell defense against infections (48), and by promoting an anti-apoptotic cellular response (49). Importantly, leptin-mediated stimulation of neutrophils requires TNF-α release from monocytes (50). In dendritic cells, leptin upregulates the production of IL-1β, IL-6, IL-12, TNF-α, and macrophage inflammatory protein (MIP)-1α, and protects dendritic cells from apoptosis. In addition, leptin downregulates IL-10 production and drives polarization of naïve T cell toward the Th1 phenotype (51). Previous studies have shown that leptin sustains cytotoxic activities of NK cells by inducing phosphorylation of STAT3 and transcription of IL-2 and perforin (52).

In the adaptive immune system, leptin promotes lymphopoiesis by stimulating maturation of CD4^+^CD8^+^ and CD4^+^CD8^−^ T cells. Leptin induces proliferation of naïve CD4^+^CD45RA^+^ T cells, but inhibits proliferation of memory CD4^+^CD45RO^+^ T cells by increasing the production of the Th1 cytokines interferon (IFN)-γ and IL-2, and suppressing the production of the Th2 cytokine IL-4 (53). Beyond this, it has been shown that leptin promotes differentiation of CD4^+^ cells into IL-17 producing Th17 cells, while suppressing formation of CD4^+^CD25^+^ T_reg_ cells, in addition to prevention of stress-induced T cell apoptosis (54–56). In malnourished individuals with extremely low BMI, a dramatic reduction in leptin levels was observed, which was associated with thymic atrophy, reduced T-cell function, and increased susceptibility to infection (57). *Ob/ob* mice, *fa/fa* rats, and humans with congenital leptin deficiency or with LEPR mutations are more susceptible to infections, which go along with a decrease in the number of total lymphocytes and circulating CD4^+^ T cells, apoptosis of thymocytes, and a shift toward a Th2 immune response. Administration of recombinant leptin substantially increased thymic cellularity and restored abnormalities in proliferating T cells and cytokine release of immune cells in *ob/ob* mice and humans with leptin deficiency (40, 56, 58, 59). In B cells, leptin induces a pro-inflammatory cytokine response through stimulation of TNF-α, IL-6, and IL-10 secretion (60). Similar to T cells, leptin limits apoptosis of B cells by enhancing expression of blc-2 and by promoting cell cycle entry via cyclin D1 (61). Reduced number of total B cells, including pre-B cells and immature B cells, and defective lymphopoiesis in *ob/ob* mice could be restored by administration of recombinant leptin (62). Taken together, it is evident that leptin has pro-inflammatory effects and exerts a crucial function in modulating immune response of both the innate and the adaptive immune system.

## Leptin, Heart Function, and Cardiovascular Diseases

**Figure 2:**
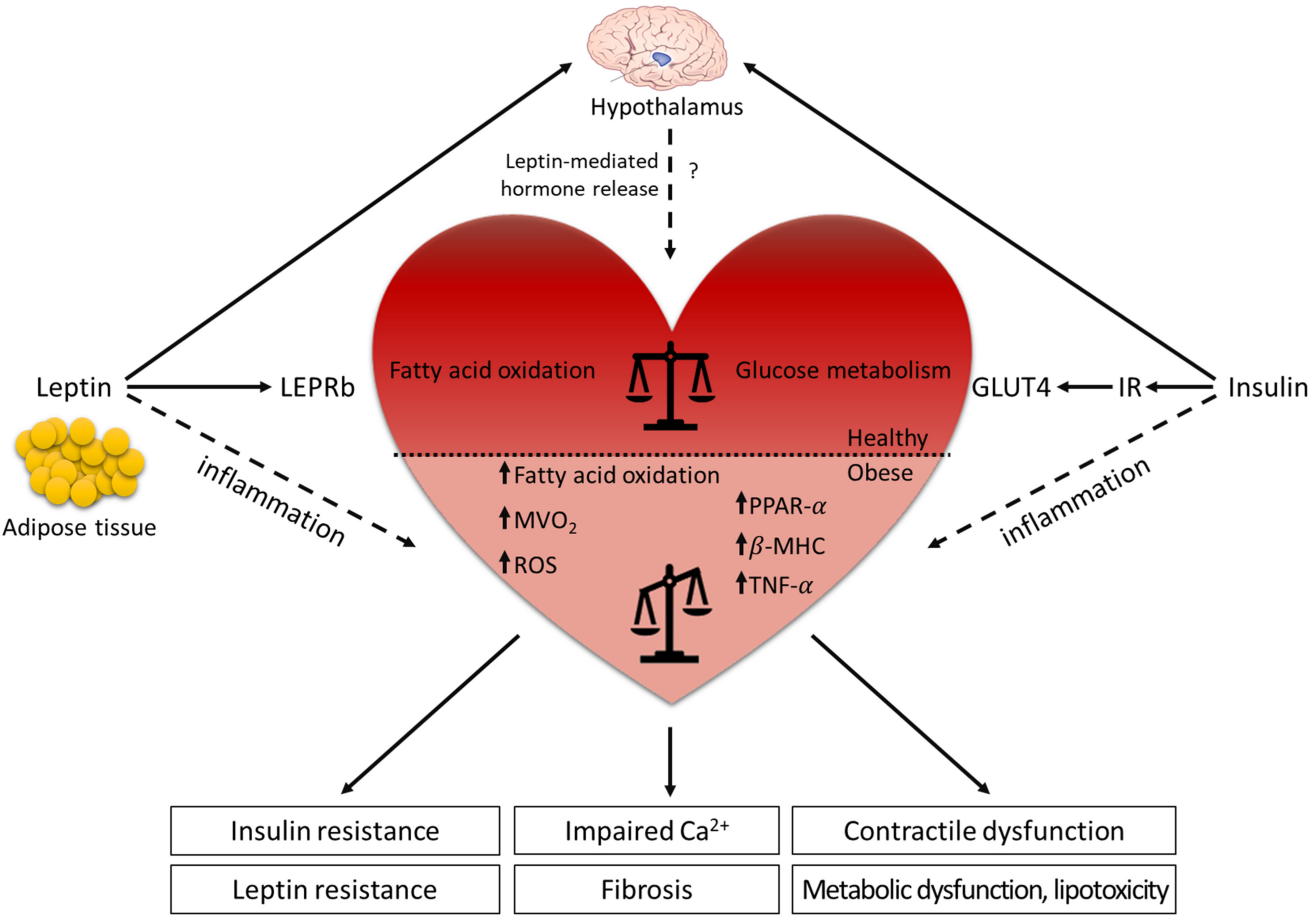
Leptin and LEPR signaling in the (dys-)regulation of the cardiovascular system. Leptin is mainly secreted from the adipose tissue and binds to the LEPR in the arcuate nucleus of the hypothalamus where it regulates food intake, energy expenditure and hormone release. The actions of leptin and insulin are interconnected and contribute to optimal metabolic control. Although leptin role in the cardiovascular system is still controversial, diabetic and obese animal models demonstrate that leptin has beneficial effects on cardiac metabolism. Under physiological condition, leptin signaling supports the balance between glucose metabolism and fatty acid oxidation in the heart, while the absence of leptin or LEPR results in the lack of the metabolic flexibility. The reduced dynamic between the energy substrates results in systemic metabolic disorders (insulin resistance, leptin resistance, metabolic dysfunction, and lipotoxicity), leading to decreased cardiac efficiency (impaired Ca^2+^ handling, contractile dysfunction, and fibrosis). In contrast, elevated leptin levels in obese patients contribute to the low-grade systemic inflammation, which increases the risk to develop cardiovascular diseases. Horizontal black line demarcates differences between the healthy heart and the heart in obesity/diabetes. LEPRb, leptin receptor b; MVO_2_, myocardial oxygen consumption; ROS, reactive oxygen species; PPAR-α, peroxisome proliferator-activated receptor α; β-MHC, myosin heavy chain β; TNF-α, tumor necrosis factor-α; GLUT4, glucose transporter 4; IR, insulin receptor.

In the cardiovascular system, the role of leptin still remains controversial. Many studies suggest that leptin is involved in the pathogenesis of chronic inflammation. In this regard, elevated leptin levels in obese patients are believed to contribute to the low-grade systemic inflammation, which makes obese individuals more susceptible to develop cardiovascular diseases (Figure 2). In addition, elevated leptin levels have been reported in patients with dilated cardiomyopathy, which are used as a biomarker for the progression of heart failure independent of immune responses (63). In a prospective study of 4,080 men, aged 60–79 years, with no diagnosed heart failure followed for 9 years, increased BMI and circulating leptin levels were used as independent predictors for the incidence of heart failure. Elevated leptin levels were associated with an increased risk of heart failure in men without pre-existing coronary heart disease, independent of BMI and potential mediators (64). In the Framingham study with 818 participants (mean age 79 years, 62% women), leptin levels were strongly associated with incidence of congestive heart failure and cardiovascular disease. After adjustment for BMI, the association of leptin with congestive heart failure was nullified, but the relation to an enhanced incidence for development of cardiovascular disease was only modestly attenuated (65). In contrast, the Multi-Ethnic Study of Atherosclerosis with randomly selected 1,905 participants (mean age 64.5 years, 50% women) without underlying cardiovascular disease revealed that leptin levels were not associated with incidence of cardiovascular disease events after adjustment for cardiovascular risk factors, BMI or waist circumference (66). Recently, the use of BMI for the risk assessment of cardiovascular disease is on a core controversy because BMI does not discriminate between body fat mass and muscle mass. It has been suggested that visceral adiposity, rather than a high BMI, is correlated with increased risk of cardiovascular diseases and diabetes (67). Based on these epidemiologic data, it remains unclear whether leptin is associated with the development of heart failure. It is possible that chronic effects of leptin may have adverse consequences on myocardial function.

Nevertheless, many studies using rodent obese and diabetic models demonstrate that leptin has beneficial effects on cardiac metabolism by supporting glucose metabolism and fatty acid oxidation. Therefore, acute effects of leptin may provide a compensatory response to cardiac insults, such as ischemia or heart failure (68).

### Animal Models for Leptin and Leptin Receptor Deficiency and Cardiovascular Diseases

Animal models have helped to understand leptin signaling in the heart (69) and both *ob/ob* and *db/db* mice show an age-dependent progression of hypertrophy, mostly effecting left ventricular (LV) mass and LV wall thickness (70, 71). In particular, *db*/*db* mice develop both systolic and diastolic dysfunctions and contractile abnormalities, but with maintenance of cardiac output by favorable loading conditions (increased preload and decreased afterload) at 12 weeks of age (72, 73). Cardiomyocyte-specific deletion of *Lepr* (*Lepr*^−/−^) in mice, in the presence of increased Cre recombinase expression, caused significant thinning of the ventricular wall and reduced ejection fraction, which was associated with impaired energy production via AMPK- and mTOR signaling. After myocardial infarction, cardiomyocyte-specific *Lepr*^−/−^ mice revealed greater cardiac dysfunction and increased morbidity compared to wild-type mice, which were associated with attenuated cardiac STAT3, PI3K, and AKT activity and mitochondrial function (74, 75). Furthermore, these studies demonstrated that impaired cardiac leptin signaling in *Lepr*^−/−^ mice resulted in metabolic inflexibility for glucose utilization in the face of cardiac stress (75). Similar to mouse models, impaired leptin signaling also contributes to the cardiac-specific phenotype in *fa/fa* and ZDF rats, as illustrated by LV hypertrophy and defects in cardiac contractility, such as reduced fractional shortening, yet these effects are less pronounced in *fa*/*fa* rats (76, 77). Moreover, *db*/*db* mice and ZDF rats are more susceptible to ischemic injury and show impaired recovery of cardiac function after myocardial infarction induced by coronary artery ligation at stages when animals have developed diabetes (69). Interestingly, while either leptin administration or caloric restriction resulted in weight loss in *ob/ob* mice, leptin infusion but not caloric restriction reversed the obesity-related cardiac remodeling process, indicating that leptin has anti-hypertrophic effects not attributable to weight loss alone (70). However, it has not been completely understood whether leptin directly affects cardiac function or acts through a leptin-regulated neurohumoral pathway (Figure 2).

Under physiological conditions, the heart uses both glucose and free fatty acids as energy substrates. In leptin- or *Lepr*-deficient rodent models, impaired cardiac performance is linked to altered myocardial substrate uptake and metabolic inflexibility due to increased vesicular fatty acid uptake and fatty acid oxidation with reduced glucose uptake and carbohydrate oxidation (Figure 2). As glucose is a more efficient energy substrate for cardiomyocytes than free fatty acids, a metabolic switch from glucose metabolism to free fatty acid oxidation results in increased myocardial oxygen consumption (MVO_2_) and decreased cardiac efficiency (ratio of cardiac work to energy input), which leads to systemic metabolic disorders (76–78). Leptin- or *Lepr*-deficient animals show elevated triglyceride levels and lipid accumulation in the myocardium that may promote lipotoxicity and directly impede cardiac contractility (79–82). The prominent effects of leptin in reducing high triglyceride levels have been confirmed in an acyl CoA synthase transgenic mouse model showing severe lipotoxic cardiomyopathy (83). Liver-specific overexpression of wild-type *Lepr* in *fa*/*fa* rats emphasized the prominent effects of leptin in reducing high triglyceride levels only in the liver but nowhere else (80). Cardiomyocyte-restricted re-expression of *Lepr* in *db/db* mice reduced cardiac triglyceride levels, without changes in the levels of plasma triglycerides, and these mice displayed lower heart weight and reduced LV wall thickness in comparison to *db*/*db* mice (84). These studies suggest that leptin might act as an anti-lipotoxic adipokine that protects the heart from harmful lipid accumulation and progression of cardiac steatosis, especially under cardiac stress conditions.

Notably, metabolic modulation to sustain glucose utilization by perinatal overexpression of the glucose transporter GLUT4 prevented cardiac dysfunction in *db*/*db* mice (72, 85). High levels of insulin and glucose normalized cardiac metabolism, restored cardiac efficiency, and improved post-ischemic recovery in *db*/*db* mice (86). Administration of a peroxisome proliferator-activated receptor γ (PPARγ) agonist to ZDF rats resulted in an increase in glucose metabolism, reduced accumulation of triacylglycerol in the myocardium, and restored cardiac function (76). Although chronic administration of *db*/*db* mice with a PPARγ agonist induced insulin sensitization, enhanced glucose oxidation, and decreased palmitate oxidation, it failed to restore cardiac function (87). These studies indicate that alterations in glucose uptake, which are caused by cardiac insulin resistance, facilitate the cardiac metabolic switch from glucose metabolism to free fatty acid oxidation, leading to increased MVO_2_ and reduced cardiac efficiency and performance. Upon ischemic injury, energy shortage in the myocardium is exacerbated, which reduces ischemic tolerance of the diabetic heart. Restoring the balance of glucose and fatty acid metabolism in cardiomyocytes following injury is of fundamental importance for functional recovery of the heart.

Increased MVO_2_ can also be a result of increased production of reactive oxygen species (ROS), which is associated with augmented fatty acid oxidation, mitochondrial uncoupling, and oxidative stress in hearts of *db/db* mice (88). Comparable to *db/db* mice, *fa*/*fa* rats display an increased oxidative stress response, as indicated by high levels of lipid peroxide and elevated activity of the superoxide dismutase (89). Another factor contributing to cardiac dysfunction in rodent models is perturbed intracellular Ca^2+^ handling. In *db*/*db* mice, systolic and diastolic Ca^2+^ levels, sarcoplasmic reticulum Ca^2+^ load, Ca^2+^ transient decay, and L-type Ca^2+^ current are all reduced while Ca^2+^ leakage from the sarcoplasmic reticulum is increased. These data demonstrate that perturbations in cardiac Ca^2+^ handling are a major cause of contractile dysfunction (90, 91). In conclusion, studies using leptin- or *Lepr*-deficient rodent models indicate that leptin acts as a metabolic, cardio-protective adipokine, particularly under ischemic conditions.

### Leptin, Cardiac Hypertrophy, and Diabetic Cardiomyopathy

Abnormal myocardial structure and impaired cardiac performance in the absence of overt cardiovascular risk factors, such as coronary artery disease, valvular heart disease, hypertension, or dyslipidemia in patients with T2DM, are described as diabetic cardiomyopathy (92). Although diabetic cardiomyopathy is usually asymptomatic in very early stages, its progression results in LV diastolic dysfunction and/or reduced ejection fraction, LV hypertrophy, and increased interstitial fibrosis (92–94). Patients with diabetic cardiomyopathy display a diastolic, but not systolic dysfunction at early stages, which gradually progress to severe diastolic heart failure with preserved ejection fraction, known as HFpEF. At later stages, these patients develop systolic dysfunction and heart failure with reduced ejection fraction (HFrEF). The prevalence of diabetic cardiomyopathy is increasing in parallel with an increase in T2DM. With an incidence of 19–26% to develop heart failure, cardiovascular remodeling processes resulting in diabetic cardiomyopathy are a major cause of disease-related deaths in patients with T2DM (92).

Clinical studies reported an association of plasma leptin levels with LV hypertrophy. In hypertensive insulin-resistant men, fasting plasma leptin levels were higher than in controls and correlated with increased myocardial wall thickness. This correlation persisted after adjustment for BMI and was independent of blood pressure (95). Moreover, Perego et al. showed that plasma leptin levels were higher in morbidly obese patients than in lean controls and were positively correlated to LV mass after adjustment for BMI. One year after bariatric surgery, obese patients showed profound weight loss and significant reductions in BMI and insulin resistance. Nevertheless, the decrease in LV mass was correlated only with a decrease in leptin levels on multivariable analyses (96). These clinical findings propose that leptin participates in the progression and development of LV hypertrophy in obese patients. Studies have also shown that leptin accounts for the increase in blood pressure associated with obesity, as obese patients with a loss-of-function mutation in leptin or LEPR displayed low blood pressure (97), and obese patients are frequently characterized by sympathetic hyperactivity and higher plasma levels of adrenaline and noradrenaline (98). Chronic leptin infusion in male rats has been shown to elevate arterial blood pressure and heart rate through the stimulation of the sympathetic nervous system activity (99). Therefore, leptin-mediated increase in heart rate and blood pressure may provoke an increase in myocardial workload, which contributes to cardiac hypertrophy over the long term.

It is important to emphasize that human patients with obesity-associated impairment of cardiac function commonly suffer from HFpEF, in combination with sodium retention, plasma volume expansion, microvascular inflammation, and atypical deposition of fibrotic tissue (100, 101). Most of these symptoms are correlated with elevated plasma leptin levels (102). Intriguingly, patients with advanced stages of chronic heart failure are hyperleptinemic, as serum levels of leptin and its soluble receptor LEPRe are increased after the adjustment for BMI (103).

The underlying mechanisms of diabetic cardiomyopathy, particularly in obesity-related T2DM, have mainly been investigated in *ob/ob* and *db/db* mice or *fa*/*fa* and ZDF rats (93). As already mentioned earlier, various metabolic disturbances are involved in the pathogenesis of diabetic cardiomyopathy, such as systemic tissue inflammation, insulin resistance, altered myocardial substrate utilization, elevated fatty acid levels, oxidative stress, and activation of the renin-angiotensin-aldosterone system. Animal models with a genetic defect in leptin signaling have helped to increase our knowledge about mechanisms contributing to the cardiac remodeling process in the diabetic, leptin-deficient heart. However, it is hard to draw any conclusions from the observations in rodent models for patients with diet-related obesity in the clinical setting, as obese patients normally display high circulating levels of leptin and are leptin resistant.

Obese patients with insulin resistance display high levels of myocardial triglycerides, which are linked to transcriptional upregulation of PPARα-regulated genes, myosin heavy chain β (β-MHC), and TNF-α, in addition to cardiac contractile dysfunction (82, 104). A study by Nyman et al. uncovered that cardiac steatosis caused by lipid oversupply in patients with metabolic syndrome was associated with LV diastolic function. Although the amount of epicardial and pericardial fat correlated with the severity of LV diastolic dysfunction, increase in myocardial triglyceride levels did not act as a mere fat deposit compared to epicardial and pericardial fat (105). *In vitro* experiments using cardiomyocytes from either patients with dilated cardiomyopathy or controls indicate that leptin acts in concert with resistin causing a TNF-α- and IL6-dependent cardiac redox stress response (63). Human and neonatal rat cardiomyocytes treated with recombinant leptin were more susceptible to progression of cardiac hypertrophy, as indicated by increased cell size, elevated matrix metalloproteinase-2 (MMP-2) activity, and endothelin-1-induced rise in ROS levels (106, 107).

Taken together, leptin exerts physiological effects that may be detrimental in states of cardiac dysfunction or heart failure. Leptin's hemodynamic effects, such as elevation of resting heart rate and blood pressure, generally increase myocardial workload via activation of the sympathetic nervous system. Leptin may act synergistically with other factors that are associated with obesity, such as hyperglycemia, inflammation, and oxidative stress, to accelerate development and progression of cardiovascular diseases (Figure 2). Recently, studies have investigated the role of sodium glucose transporter 2 (SGLT2) inhibitors, such as empagliflozin, on modulating cardiac alterations in obesity-related T2DM. As leptin is implemented to regulate the expression of SGLT2 in renal tubules through activation of the sympathetic nervous system and the renin-angiotensin system, SGLT2 inhibitors have been shown to reverse leptin-induced renal sodium and glucose retention and inflammatory response in adipocytes by reducing secretion of leptin and limiting its paracrine effects on cardiac and renal metabolism (108). SGLT2 inhibitors are known not only to improve cardiac function and metabolism, but also to promote weight loss by reducing visceral fat depots and to attenuate the obesity-induced inflammatory response and insulin resistance by modulating the activity of M2 macrophages (109, 110). Most importantly, SGLT2 inhibitors significantly reduced the number of deaths arising from T2DM-associated complications on the cardiovascular system (111).

### Leptin, Cardiac Fibrosis, and Vascular Dysfunction

Leptin-mediated aldosterone production is considered a novel mechanism of obesity-associated endothelial dysfunction and cardiac fibrosis, which impair myocardial relaxation and thereby contribute to cardiovascular disease (112). This explains why the aldosterone antagonist spironolactone is particularly effective in reducing morbidity and mortality in patients with obesity-related HFpEF (113). Moreover, spironolactone improved LV function and reduced levels of circulating procollagen in obese patients without any other comorbidities (114). The correlation between leptin and aldosterone levels was also observed in cardiac myofibroblasts of high-fat diet-fed rats. Here, the aldosterone antagonist eplerenone reduced the leptin-induced increase in protein levels of pro-fibrotic factors collagen I, TGFβ, connective tissue growth factor, and galectin-3 as well as the levels of both total and mitochondrial ROS (115).

In a study of 294 healthy adolescents (aged 13–16 years) with a broad ranged BMI, an elevation in leptin levels was associated with impaired arterial distensibility, independent of metabolic and inflammatory disturbances associated with obesity (116). Moreover, increased plasma leptin levels were positively correlated with the total number of stenotic coronary arteries in patients with coronary artery disease (117). These data indicate that leptin plays a key role in the development of vascular dysfunction. This is supported by studies showing that *ob/ob* mice did not develop any atherosclerotic lesions or plaques despite being fed with a chow diet containing 2% cholesterol over 2 months, which is contrary to expectations in obese, diabetic individuals with hypercholesterolemia (41, 118). *In vitro* studies using human umbilical vein endothelial cells demonstrate that leptin induces chronic oxidative stress in endothelial cells, which may promote atherogenic processes and contribute to the development of vascular pathology (119). Leptin may stimulate proliferation and migration of vascular smooth muscle cells (120) and induce the calcification of vascular cells (121), thereby contributing to the formation and development of vascular lesions. In addition, it is generally accepted that leptin-mediated release of endothelial nitric oxide (NO) causes vasodilatation, which opposes the pressor effect of sympathoexcitatory activity induced by leptin (122, 123). However, in patients with obesity and T2DM, hyperleptinemia caused a reduction of NO bioavailability and attenuated NO-dependent vasodilation, which contributes to vascular dysfunction (124).

## Conclusions

Overall, leptin has emerged as an adipocyte-derived factor with pleiotropic effects in the nutritional and metabolic state, in modulating immune responses and inflammation, and in controlling cardiovascular functions (Figure 2). Animal studies using leptin- or Lepr-deficient rodents demonstrate that the cardiac dysfunction is associated with (i) a metabolic switch from glucose metabolism to fatty acid oxidation, promoting lipotoxicity, (ii) systemic inflammation, (iii) insulin resistance, and (iv) activation of the renin-angiotensin-aldosterone system. However, the alteration of lipid profile in leptin- or Lepr-deficient animal models cannot fully recapitulate the phenotypes of patients with T2DM. Leptin resistance in obese or T2DM patients adversely affects the heart's response to stress conditions and promotes cardiac remodeling as a result of impaired cardiac metabolism, increased fibrosis, vascular dysfunction, and enhanced inflammation, all contributing to impairment of cardiac function. Even after decades of research on rodents and clinical studies, existing differences in the underlying mechanisms and pathways between animal models and patients with obesity and T2DM make data interpretation challenging. This hampers the development of new strategies to treat patients with diabetic cardiomyopathy (or HFpEF).

Since leptin is a critical regulator of the cardiovascular system, new strategies targeting leptin's action precisely in the cardiovascular system in normal and diseased states might be important. Future research is required to understand the precise role of leptin directly in different cardiac cell types (e.g., cardiomyocytes, endothelial cells or fibroblasts), which may open up new avenues for the treatment of obesity- and diabetes-associated cardiomyopathy. Disease modeling using human induced pluripotent stem cells combined with the CRISPR/Cas9 technique may allow us to study and better understand abnormal leptin signaling in cardiomyocytes as well as in endothelial cells *in vitro*, which might cause obesity- and T2DM-associated cardio-metabolic remodeling processes. Consequently, a shift from leptin- and Lepr-deficient rodent models to human-based models will be of great importance to overcome the translational barrier and to develop innovative therapeutic approaches.

## Author Contributions

MP, AS, and KG conducted a review of the literature and wrote the first draft of the review. KG contributed to conception and design of the article and finalized the review. All authors read and approved the final manuscript.

## Conflict of Interest

The authors declare that the research was conducted in the absence of any commercial or financial relationships that could be construed as a potential conflict of interest.
